# Supplementary material for: Pattern, causes and functional outcome of peripheral neuropathies in the Amazon region
Source: PLoS One. 2024 Dec 20;19(12):e0315760. doi: 10.1371/journal.pone.0315760 (PMC11661610; doi:10.1371/journal.pone.0315760)
Supplement: S1 Table — (DOCX) [file pone.0315760.s001.docx]

**S1 Table. Number at risk and number censored in Kaplan-Meir survey analysis.**

|  | **T=0** | | **T=2** | | **T=4** | | **T=6** | | **T=8** | |
| --- | --- | --- | --- | --- | --- | --- | --- | --- | --- | --- |
|  | Nb at risk(Nb censored) | Death | Nb at risk(Nb censored) | Death | Nb at risk(Nb censored) | Death | Nb at risk(Nb censored) | Death | Nb at risk(Nb censored) | Death |
| Diabetes | 346 (0) | 7 | 232(178) | 10 | 111 (252) | 7 | 37 (312) | 1 | 1(321) | 0 |
| Infections | 11(0) | 1 | 7(4) | 0 | 6 (5) | 0 | 5 (8) | 0 | 1(10) | 0 |
| Toxic | 120(0) | 8 | 64(70) | 8 | 27 (84) | 2 | 10 (97) | 0 | 3(102) | 0 |
| Unlabeled | 17 (0) | 1 | 11 (8) | 0 | 4 (14) | 0 | 0 | 0 | 0 | 0 |
| Multiple causes | 17(0) | 0 | 16(4) | 1 | 10(10) | 0 | 4(16) | 0 | 0 | 0 |
| Others | 45 (0) | 1 | 24(24) | 0 | 15(36) | 0 | 7(39) | 2 | 1(42) | 0 |
